# Belowground Carbon Efficiency for Nitrogen and Phosphorus Acquisition Varies Between *Lolium perenne* and *Trifolium repens* and Depends on Phosphorus Fertilization

**DOI:** 10.3389/fpls.2022.927435

**Published:** 2022-06-24

**Authors:** Jiayu Lu, Jinfeng Yang, Claudia Keitel, Liming Yin, Peng Wang, Weixin Cheng, Feike A. Dijkstra

**Affiliations:** ^1^CAS Key Laboratory of Forest Ecology and Management, Institute of Applied Ecology, Chinese Academy of Sciences, Shenyang, China; ^2^School of Life and Environmental Sciences, Sydney Institute of Agriculture, The University of Sydney, Sydney, NSW, Australia; ^3^National Engineering Laboratory for Efficient Utilization of Soil and Fertilizer Resources, College of Land and Environment, Shenyang Agricultural University, Shenyang, China; ^4^Environmental Studies Department, University of California, Santa Cruz, Santa Cruz, CA, United States

**Keywords:** belowground carbon allocation, biological nitrogen fixation, carbon allocation for nutrient uptake, ^13^C-labeling, rhizosphere priming effect

## Abstract

Photosynthetically derived carbon (C) is allocated belowground, allowing plants to obtain nutrients. However, less is known about the amount of nutrients acquired relative to the C allocated belowground, which is referred to as C efficiency for nutrient acquisition (CENA). Here, we examined how C efficiency for nitrogen (N) and phosphorus (P) acquisition varied between ryegrass (*Lolium perenne*) and clover (*Trifolium repens*) with and without P fertilization. A continuous ^13^C-labeling method was applied to track belowground C allocation. Both species allocated nearly half of belowground C to rhizosphere respiration (49%), followed by root biomass (37%), and rhizodeposition (14%). With regard to N and P, CENA was higher for clover than for ryegrass, which remained higher after accounting for relatively low C costs associated with biological N_2_ fixation. Phosphorus fertilization increased the C efficiency for P acquisition but decreased the C efficiency for N acquisition. A higher CENA for N and P in clover may be attributed to the greater rhizosphere priming on soil organic matter decomposition. Increased P availability with P fertilization could induce lower C allocation for P uptake but exacerbate soil N limitation, thereby making N uptake less C efficient. Overall, our study revealed that species-specific belowground C allocation and nutrient uptake efficiency depend on which nutrient is limited.

## Introduction

Belowground carbon (C) allocation by plants is an important driver for plant nutrient acquisition. In a global synthesis, Pausch and Kuzyakov (2018) reported that grassland species allocated on an average of 33% of gross primary productivity belowground to root biomass, rhizosphere respiration, and rhizodeposition. This belowground C is tightly associated with plant nutrient acquisition through various strategies, such as generating fine roots or root hairs, forming symbiotic associations with nitrogen (N)-fixing bacteria or mycorrhizal fungi, or stimulating microbial activity to mobilize nutrients from soil organic matter using root exudates (Lambers et al., 2008; Richardson et al., 2009; Zhu et al., 2014). Modeling studies have indicated that variation in C allocation for nutrient acquisition between N-fixing and non-fixing plants, or between arbuscular and ectomycorrhizal plants, is helpful for understanding their competitive advantages and how this relates to their abundance and productivity (Fisher et al., 2010; Brzostek et al., 2014). However, empirical research on how much total C plants allocate belowground to obtain nutrients is rare.

To assess the C efficiency associated with nutrient acquisition, we introduce a new parameter, i.e., C efficiency for nutrient acquisition (CENA), which we define as the amount of nutrients acquired relative to C allocated belowground (Wang et al., 2022). In most studies, belowground C allocation is primarily based on measures of root production, thus ignoring other C pathways such as allocation to root respiration, root exudates, and symbiotic relationships, which are extremely difficult to quantify (Vicca et al., 2012; Pausch and Kuzyakov, 2018; Keller et al., 2021). This assessment, without considering other C pathways, may underestimate total belowground C allocation, hindering an accurate understanding of CENA. ^13^C-labeling methods provide us with the opportunity to quantify root respiration, root exudates, and symbiotic microbial respiration, which have been successfully applied in previous studies (Schmitt et al., 2013; Ven et al., 2019). Compared to traditional methods, the isotope tracer method permits us to consider all these belowground C allocation pathways. As rhizosphere respiration and rhizodeposition may account for a large proportion of the total C allocated belowground (Pausch and Kuzyakov, 2018), accounting for all belowground C allocation pathways will be required to accurately estimate CENA.

Belowground C allocation and CENA can vary greatly among plant species due to differences in root architecture and morphological traits, root exudates, mycorrhizal association, and the capacity of biological N_2_ fixation (de Neergaard and Gorissen, 2004; Schmitt et al., 2013; Keller and Phillips, 2019). For example, legumes allocated more C to rhizosphere respiration compared to grasses, because of the extra energy and C demand for biological N_2_ fixation by symbiotic rhizobia (Warembourg et al., 2003), while grasses may spend more C on dense fine roots or high rates of rhizodeposition for enhancing nutrient mobilization and uptake from the soil (Schmitt et al., 2013). The interspecific difference in belowground C allocation patterns will trigger different responses in nutrient acquisition, thereby influencing CENA between legumes and grasses.

Soil nutrient availability may also be an important factor influencing belowground C allocation and CENA. Using economic principles, it can be expected that nutrients become more C expensive for plants when their availability is low (Bloom et al., 1985). Most plants are limited by N, phosphorus (P), or both (Harpole et al., 2011; Du et al., 2020), and the amount of C that plants allocate belowground may strongly depend on which nutrient is limiting their growth. Although plant demand for P is lower than for N, belowground C allocation for P uptake may be higher than for N given that soil P availability is usually much lower and less mobile compared to N (Vitousek et al., 2010). Plants secrete carboxylates to liberate inorganic P from mineral surfaces, or produce phosphatase extracellular enzymes to increase P mobilization through hydrolysis, when P availability to plants is limited (Lambers et al., 2008; Richardson et al., 2011; Wen et al., 2019). Furthermore, plants may increase belowground C allocation to support arbuscular mycorrhizal fungi to enhance P uptake under low P conditions (Smith et al., 2011; van der Heijden et al., 2015; Ven et al., 2019). Therefore, plants may allocate more belowground C to root exudates or rhizosphere respiration when plants are limited by P. Due to their capacity to fix N_2_ from the atmosphere, the growth of legumes is more likely to be P-limited (Png et al., 2017), and belowground C allocation and CENA in legumes may therefore be more sensitive to P availability in soil compared to grasses.

Here, we assessed belowground C allocation and C efficiency for N and P acquisition in ryegrass (*Lolium perenne* L., C_3_ grass) and clover (*Trifolium repens* L., legume) with and without P fertilization based on the same greenhouse experiment in Lu et al. (2020). By continuously labeling plants with CO_2_ depleted in ^13^C, we were able to quantify different components of belowground C allocation (root biomass, rhizosphere respiration, and rhizodeposition). We further used a ^15^N natural abundance method to estimate biological N_2_ fixation in clover and finally assessed CENA by comparing belowground C allocation to nutrient content in plant biomass after 58 days of growth. The objectives of this study were to (1) compare the difference in C efficiency for N (CENA_N_) and for P (CENA_P_) between ryegrass and clover and (2) assess how P fertilization affects CENA_N_ and CENA_P_ in ryegrass and clover. We hypothesized that (1) clover would have a higher C efficiency for N (CENA_N_) compared to ryegrass because plant N acquisition through biological N_2_ fixation is usually more C efficient compared to uptake from the soil (Fisher et al., 2010); (2) P fertilization would increase CENA_N_ in clover because P fertilization would increase biological N_2_ fixation and reduce belowground C allocation associated with P uptake from the soil. However, P fertilization would increase N limitation in ryegrass, lowering CENA_N_; and (3) C efficiency for P (CENA_P_) would be lower in clover because biological N_2_ fixation would cause it to be more limited by P compared to ryegrass; for the same reason, P fertilization would increase CENA_P_ more in clover than in ryegrass.

## Materials and Methods

### Experimental Design

Top soil (0–15 cm depth) was collected from a grassland at John Bruce Pye Farm in Camden, NSW, Australia (33°56′42″ S, 150°40′30″ E). The soil is a red-brown chromosol (Isbell, 2002) (or Alfisol based on USDA Soil Taxonomy), with a pH of 5.4, 34% sand, 31% silt, and 35% clay. The δ^13^C of soil organic C was −23.06‰, and the organic C, total N, and total P concentrations were 28.8, 2.5, and 0.15 mg g^–1^, respectively. The concentrations of soil mineral N (2 M KCl extraction) and available P (0.03 M NH_4_F and 0.025 M HCl) were 58.0 and 8.7 mg kg^–1^, respectively. Mesocosms consisted of bottom-capped polyvinyl chloride (PVC) pots (diameter 15 cm, height 20 cm) and sieved (4 mm) grassland soil (equivalent to 3.20 kg of oven-dried soil). After adjusting soil moisture content to 70% water-holding capacity (21% gravimetric soil moisture content), a modified Hoagland solution with macro- and micro-nutrients was added to all mesocosms [(NH_4_)_2_SO_4_ 23.8, KNO_3_ 25.7, Ca(NO_3_)_2_⋅4H_2_O 11.9, MgCl_2_⋅6H_2_O 16.4, H_3_BO_3_ 0.08, ZnSO_4_⋅7H_2_O 0.2, CuSO_4_⋅5H_2_O 0.02, FeSO_4_⋅7H_2_O 0.25, and MnCl_2_⋅4H_2_O 0.3 g m^–2^]. The P was applied to the treatment with P as a KH_2_PO_4_ and K_2_HPO_4_ solution with an adjusted ratio to obtain a similar pH to the soil (4 g of P m^–2^), while for the treatment without P, a KCl solution was applied to eliminate the introduced K fertilization effect. These mesocosms (with and without P) were planted with either ryegrass (*Lolium perenne* L.) or clover (*Trifolium repens* L.) or were left unplanted (control). These two plant species are widely used to improve pastures in many temperate regions of the world. Besides the capacity of fixing N_2_ from the atmosphere, ryegrass and clover also differ greatly in root morphological and architectural traits (Table 1), mycorrhizal infection (Zhu et al., 2000), and quantity and quality of root exudates (Lu et al., 2020), thereby showing different strategies of carbon allocation and nutrient acquisition. Six treatments were replicated four times. After germinating, each planted mesocosm was thinned to 20 plants.

**TABLE 1:** Mean diameter (MD), specific root length (SRL), specific root surface area (SRA), root tissue density (RTD), and root length density (RLD) of ryegrass and clover with and without P addition.

Treatment	MD, mm	SRL, m g^–1^	SRA, cm^2^ g^–1^	RTD, g cm^–3^	RLD, m cm^–3^
Ryegrass − P	0.23 ± 0.01b	220 ± 24a	1561 ± 119a	0.11 ± 0.01b	0.32 ± 0.04a
Ryegrass + P	0.24 ± 0.01b	208 ± 11a	1533 ± 58a	0.11 ± 0.01b	0.31 ± 0.03a
Clover − P	0.29 ± 0.01a	87 ± 7b	790 ± 42b	0.17 ± 0.01a	0.11 ± 0.01b
Clover + P	0.29 ± 0.01a	87 ± 4b	801 ± 31b	0.17 ± 0.01a	0.10 ± 0.01b
**ANOVA (*p*-values)**					
Species	< 0.001	< 0.001	< 0.001	< 0.001	< 0.001
P	0.601	0.676	0.906	0.61	0.76
Species × P	0.684	0.649	0.792	0.956	0.903

*Values are shown as mean ± SE (n = 4). Two-way ANOVA p-values are shown.*

This experiment lasted for 58 days in a controlled environmental facility at the Centre for Carbon, Water, and Food, University of Sydney, Camden (NSW). During the experimental period, the air temperature was kept at 25°C during the day and at 15°C during the night. The relative air humidity was kept at 60%, and artificial lights (Heliospectra, LX602C, 600 W, ∼1 mmol m^–2^ s^–1^) went on for 12 h every day. The concentration of CO_2_ was set at 800 ppm by injecting ^13^C-depleted CO_2_ into the chamber. This was needed to reduce the δ^13^C value of CO_2_ to a desired level and also to promote plants to grow faster, so that the labeled ^13^C signature could be detected in different pools or fluxes of belowground C allocation during a short cultivation period. Although a high level of CO_2_ concentration would affect plant photosynthesis and thereby nutrient uptake dynamics, we are not aware that it would cause significant bias when comparing the treatment effects (species and P) under the same CO_2_ concentration. Moreover, our study provides novel insights into a mechanistic understanding of plant–soil interactions and is relevant given that global atmospheric CO_2_ concentrations may reach 800 ppm or higher by the end of this century (Collins et al., 2013). We further note that we have used this method successfully in the past (e.g., Dijkstra and Cheng, 2007a; Canarini and Dijkstra, 2015). The δ^13^C value of CO_2_ was maintained at −20 ± 0.3‰ (mean ± standard deviation) throughout the experiment (measured on a G2131-i Analyzer, Picarro, Santa Clara, CA, United States). More details about continuous ^13^C-labeling method were reported in Lu et al. (2020). All mesocosms were randomly placed in the chamber and watered every 2 days to maintain soil moisture content at 70% water-holding capacity. The distribution of these mesocosms was randomly rotated every week to eliminate potential effects caused by spatial differences in light levels within the chamber.

### Measurements

Total soil respiration was measured using a gas chamber method on 30, 44, and 58 days after planting (see full details in Lu et al., 2020). Briefly, at each gas sampling time, shoots were clipped at 1 cm above the soil surface and a non-transparent PVC chamber was sealed to each mesocosm. After removing initial CO_2_ inside the mesocosm and chamber by circulating air inside the mesocosm through a soda lime column, a 12 mL gas sample was taken from the septum of the chamber at 0 h (T0), 1 h (T1), and 2 h (T2), respectively. These gas samples (T0, T1, and T2) were measured for CO_2_ concentration and δ^13^C on a Delta V advantage isotope ratio mass spectrometer (IRMS) coupled to a Gasbench (Thermo Fisher Scientific, Bremen, Germany). As plants were continuously labeled with depleted ^13^C-CO_2_ (δ^13^C = −20‰), we were able to separate root-derived CO_2_ (root respiration and microbial respiration of rhizodeposits) from soil-derived CO_2_. At the end of the experiment, root samples were carefully picked from the mesocosm and soil samples were homogenized. The clipped shoots at each sampling time, hand-picked roots, and homogenized soil were measured for C%, N%, δ^13^C, and δ^15^N on a Delta V advantage IRMS coupled to a Conflo IV and Flash HT (Thermo Fisher Scientific, Bremen, Germany). The plant samples were also measured for P concentration on the UV–VIS spectrophotometer (UVmini-1240), following the protocol described by Jackson (1958).

### Calculations

Belowground C allocation includes C for root growth (root biomass C), rhizosphere respiration (root-derived CO_2_), and rhizodeposition (root-derived SOC). For clover, rhizosphere respiration also includes a C cost for biological N_2_ fixation. We calculated rhizosphere respiration at each sampling date using a mass balance method based on δ^13^C signatures of CO_2_ in planted and unplanted mesocosms (Lu et al., 2020) as follows:


(1) $${\text{C}}_{\text{root}}={\text{C}}_{\text{total}}\times ({\text{δ}}^{\text{13}}{\text{C}}_{\text{total}}–{\text{δ}}^{\text{13}}{\text{C}}_{\text{soil}})/({\text{δ}}^{\text{13}}{\text{C}}_{\text{root}}–{\text{δ}}^{\text{13}}{\text{C}}_{\text{soil}})$$

(2) $${\text{C}}_{\text{soil}}={\text{C}}_{\text{total}}–{\text{C}}_{\text{root}}$$

where C_total_, C_soil_, and C_root_ are total belowground CO_2_, soil-derived CO_2_, and root-derived CO_2_ in planted mesocosms, respectively. δ^13^C_total_ is the measured δ^13^C value of total belowground CO_2_ in planted treatments. δ^13^C_soil_ is the mean δ^13^C value of soil respiration in the unplanted control. δ^13^C_root_ is the δ^13^C value of root-derived CO_2_ in planted treatments, which was calculated based on the δ^13^C value of root tissue corrected by a fractionation factor of root-derived CO_2_ relative to root tissue (−1.74‰ for grass and −2.67‰ for legume; Werth and Kuzyakov, 2010). We calculated the rhizosphere priming effect as the difference in soil-derived CO_2_ between planted and unplanted control treatments (Lu et al., 2020).

Rhizosphere respiration during the whole 58-day experiment was then calculated using a linear extrapolation method based on the root-derived CO_2_ at three sampling dates (30, 44, and 58 days after planting). It is noted that clipping before trapping belowground CO_2_ may cause a decrease in root-derived CO_2_ (Shahzad et al., 2012). Root-derived CO_2_ was only measured during day time in this study. Root-derived CO_2_ at night in wheat was sometimes lower than during the day, but at other times, night-time root-derived CO_2_ was similar to day-time rhizosphere respiration (Kuzyakov and Cheng, 2001). Furthermore, we noted that the respiration of crowns and a negligible amount of shoot biomass were included in our measured rhizosphere respiration. Therefore, the calculated cumulative rhizosphere respiration during the entire experiment may have been somewhat overestimated, but unfortunately, we were unable to quantify this.

We calculated new root-derived SOC formed during the experiment (C_new_) based on δ^13^C signatures of soil organic C at the start and end of the experiment in planted mesocosms (Dijkstra and Cheng, 2007b) as follows:


(3) $${\text{C}}_{\text{new}}={\text{C}}_{\text{end}}\times ({\text{δ}}^{\text{13}}{\text{C}}_{\text{initial}}–{\text{δ}}^{\text{13}}{\text{C}}_{\text{end}})/({\text{δ}}^{\text{13}}{\text{C}}_{\text{initial}}–{\text{δ}}^{\text{13}}{\text{C}}_{\text{root}})$$

where C_initial_ and C_end_ are the total amount of soil organic C at the beginning and end of the experiment, respectively. δ^13^C_initial_ is the δ^13^C value of C_initial_, δ^13^C_end_ is the δ^13^C value of C_end_, and δ^13^C_root_ is the δ^13^C value of root biomass.

Carbon efficiency for nutrient acquisition was calculated as plant nutrient content divided by belowground C allocation. Plant N and P contents were calculated by multiplying tissue biomass with tissue N and P concentration. Considering the C cost for biological N_2_ fixation in clover, we calculated CENA associated with belowground C allocation for plant nutrient uptake from the soil only, by subtracting C used for biological N_2_ fixation from the total belowground C allocation (both for CENA_N_ and CENA_P_) and subtracting biologically fixed N from the total plant N content (for CENA_N_) in clover treatments as follows:


(4) $${\text{CENA}}_{\text{N}}=\left({\text{N}}_{\text{plant}}–{\text{N}}_{\text{fix}}\right)/\left({\text{C}}_{\text{below allocation}}–{\text{C}}_{\text{biological N fixation}}\right)$$

(5) $${\text{CENA}}_{\text{P}}={\text{P}}_{\text{plant}}/\left({\text{C}}_{\text{below allocation}}–{\text{C}}_{\text{biological N fixation}}\right)$$

where N_plant_ and P_plant_ are the N and P contents in the total plant biomass (shoots and roots) after day 58 plus the N and P contents in shoot biomass clipped on day 30 and 44, respectively. N_fix_ is the biologically fixed N in clover, C_below allocation_ is the total belowground C allocation, and C_biological N fixation_ is the C used for biological N_2_ fixation. Carbon costs associated with biological N_2_ fixation in legumes are relatively constant, ranging between 8 and 12 g C g^–1^ fixed N, depending on soil temperature (Fisher et al., 2010). Here, we used a value of 8 g C g^–1^ fixed N as the C cost for biological N_2_ fixation in clover at 20°C. Biologically fixed N in clover was calculated using the ^15^N natural abundance method (Mia et al., 2018) as follows:


(6) $${\text{N}}_{\text{fix}}={\text{N}}_{\text{clover}}\times ({\text{δ}}^{\text{15}}{\text{N}}_{\text{ryegrass}}–{\text{δ}}^{\text{15}}{\text{N}}_{\text{clover}})/({\text{δ}}^{\text{15}}{\text{N}}_{\text{ryegrass}}–{\text{δ}}^{\text{15}}{\text{N}}_{\text{bnf}})$$

where N_clover_ is the N content in clover tissues, δ^15^N_ryegrass_ and δ^15^N_clover_ are the δ^15^N values of ryegrass (used as a reference plant) and clover tissues, respectively, and δ^15^N_bnf_ is the δ^15^N value of N-fixing plants completely relying on biological N_2_ fixation (without N uptake from soil), which was estimated as −1.527‰ for clover (Mia et al., 2018).

### Statistical Analyses

Two-way ANOVA was applied to test the main and interactive effects of plant species and P fertilization on root biomass C, rhizosphere respiration, rhizodeposition, belowground C allocation, plant tissue δ^15^N, biologically fixed N, and CENA. The *post hoc* Tukey’s honest significant difference (HSD) test was used to compare variables among ryegrass, ryegrass with P fertilization, clover, and clover with P fertilization treatments. Differences at *p* < 0.05 were considered significant, while differences between *p* > 0.05 and *p* < 0.1 were considered marginally significant. All statistical analyses were performed using the SPSS 20.0 (IBM SPSS Statistics 20, Armonk, United States).

## Results

### Belowground C Allocation

**FIGURE 1:**
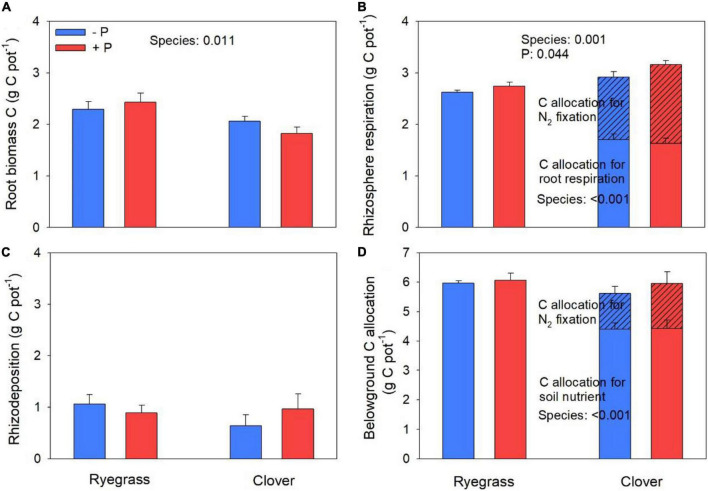
Root biomass C **(A)**, rhizosphere respiration **(B)**, rhizodeposition **(C)**, and total belowground C allocation **(D)** of ryegrass and clover with and without P addition. For rhizosphere respiration and total belowground C allocation, allocation was separated into C allocation for biological N fixation (shaded bars) and for nutrient uptake from the soil (non-shaded bars). Sub-legend shows ANOVA *p*-values. Error bar indicates one standard error of the mean (*n* = 4).

Ryegrass showed larger root biomass C than clover (Figure 1A); clover showed larger rhizosphere respiration than ryegrass (Figure 1B); and the two species did not differ in rhizodeposition (Figure 1C). Due to the contrasting patterns of root biomass and rhizosphere respiration, there was no significant difference in belowground C allocation between the two species (Figure 1D). Both ryegrass and clover allocated more belowground C to rhizosphere respiration (45 and 53%) and less to root biomass (40 and 34%) and rhizodeposition (15 and 13%) (Figure 1). For clover, the C cost for biological N fixation accounted on an average for 45% of rhizosphere respiration and 24% of total belowground C allocation, respectively. When excluding C cost for biological N_2_ fixation, clover showed lower rhizosphere respiration (Figure 1B) and less belowground C allocation than ryegrass (Figure 1D). P fertilization increased rhizosphere respiration (on an average by 6%; Figure 1B) but did not significantly influence the total belowground C allocation of both species (Figure 1D).

### Plant Nutrient Acquisition and Carbon Efficiency for Nutrient Acquisition

**FIGURE 2:**
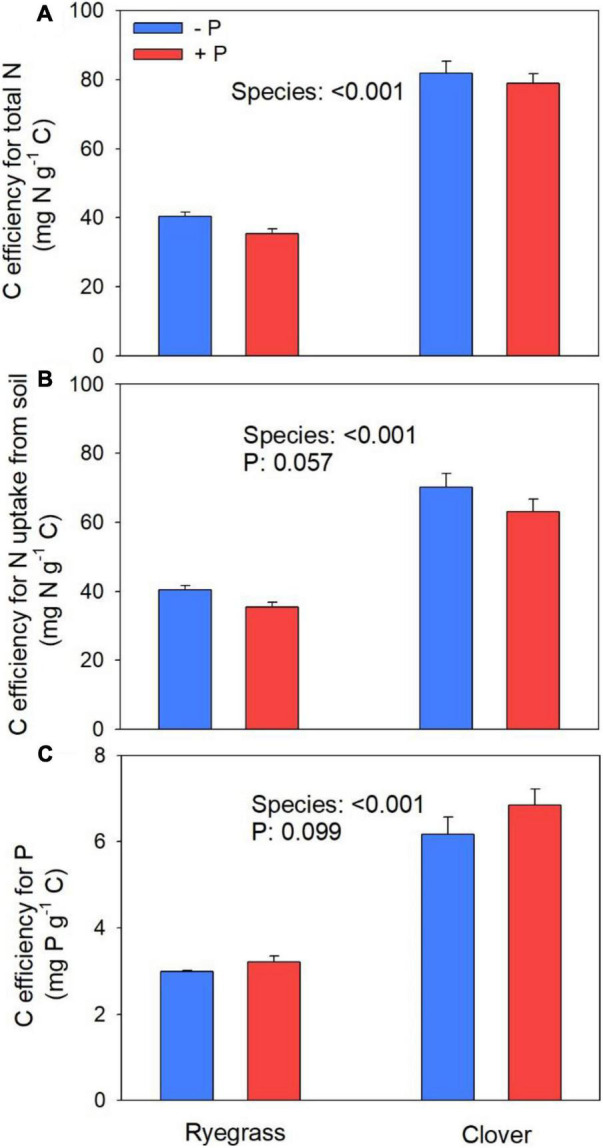
Belowground C efficiency for N acquisition (CENA_N_) including **(A)** and excluding **(B)** biological N_2_ fixation and associated C allocation, and belowground C efficiency for P acquisition (CENA_P_) excluding C used for biological N fixation **(C)** for ryegrass and clover with and without P addition. Sub-legend shows ANOVA *p*-values. Error bar indicates one standard error of the mean (*n* = 4).

Plant N and P contents were reported in Lu et al. (2020). Briefly, clover had higher plant N and P contents than ryegrass (104 and 53% higher, respectively). On average, 37% of plant N in clover was biologically fixed by rhizobia from the atmosphere (Table 2). After subtracting biologically fixed N, non-fixed N in clover was still higher than in ryegrass (28%). Phosphorus fertilization significantly increased plant P content in both species (10%), marginally increased biologically fixed N in clover (27%, Table 2) and significantly decreased non-fixed N in both species (11%). CENA_N_ (including C cost for biological N_2_ fixation) was higher for clover than for ryegrass (Figure 2A). When excluding C cost for biological N_2_ fixation, clover still showed a higher CENA_N_ than ryegrass (Figure 2B), while CENA_P_ was also higher for clover (Figure 2C), indicating that clover obtained more N and P from soil with less belowground C. Phosphorus addition increased CENA_P_ (Figure 2C) but decreased CENA_N_ in both species (Figure 2B), although the effects were marginally significant.

**TABLE 2:** Plant δ^15^N values in shoot and root biomass of ryegrass and clover, and biologically fixed N in clover with and without P fertilization (T1, Day 30; T2, Day 44; T3, Day 58).

Treatments	Plant δ^15^N				Fixed N (mg pot^–1^)
	T1-shoot δ^15^N	T2-shoot δ^15^N	T3-shoot δ^15^N	Root δ^15^N	
Ryegrass − P	2.57 ± 0.06ab	2.35 ± 0.12a	1.59 ± 0.07a	1.63 ± 0.22a	0
Ryegrass + P	2.28 ± 0.08b	1.55 ± 0.13bc	1.06 ± 0.12b	1.31 ± 0.04ab	0
Clover − P	3.08 ± 0.31a	1.97 ± 0.14ab	−0.43 ± 0.06c	1.02 ± 0.17ab	151 ± 12.2
Clover + P	2.69 ± 0.1ab	1.07 ± 0.15c	−1.01 ± 0.11d	0.77 ± 0.13b	191 ± 16.4
**ANOVA (*p*-values)**					
Species	0.016	0.007	< 0.001	0.003	–
P	0.065	< 0.001	< 0.001	0.088	–
Species × P	0.770	0.712	0.771	0.836	–

*Values are shown as mean ± SE (n = 4). Two-way ANOVA p-values are shown.*

## Discussion

Half of the total belowground C allocation was allocated to rhizosphere respiration (including root respiration and rhizosphere microbial respiration of rhizodeposits), suggesting that autotrophic respiration plays an important role in plant belowground C allocation and the total soil CO_2_ efflux (Hopkins et al., 2013). Rhizodeposition remaining in soil accounted for the lowest fraction of belowground C allocation (an average of 14%), but actual rhizodeposition rates must be higher considering that most of the rhizodeposition is lost as CO_2_ *via* rapid microbial decomposition (Pausch et al., 2013; Pausch and Kuzyakov, 2018; Liu et al., 2019), which in our case was included in rhizosphere respiration. Root biomass accounted for 37% of total belowground C allocation, which was smaller than rhizosphere respiration and rhizodeposition. These results suggest that the assessment of belowground C allocation based only on root biomass is somewhat biased and that rhizosphere respiration and rhizodeposition cannot be ignored. We acknowledge that our estimates of rhizosphere respiration were based on only three flux measurements and that they were done during the day only, and as such have the greatest uncertainty that needs further investigation. Nevertheless, the distribution of belowground C among different pools or fluxes is to some degree consistent with the C allocation patterns for grassland species synthesized by Pausch and Kuzyakov (2018).

Although clover allocated less C to root biomass, it showed greater rhizosphere respiration than ryegrass, possibly because biological N_2_ fixation by clover requires C. Indeed, when accounting for C allocation toward biological N_2_ fixation and assuming that this C would eventually be respired as CO_2_, there was a larger rhizosphere respiration in ryegrass than in clover instead (Figure 1B). Thus, C allocation toward biological N_2_ fixation was an important component of rhizosphere respiration for clover. Previous studies also suggested that legumes had a higher demand for assimilated C as indicated by higher rhizosphere respiration than grasses (Warembourg et al., 2003; Schmitt et al., 2013). Furthermore, we estimated that the C cost for biological N_2_ fixation was about 8 g C g^–1^ N (C cost at 20°C) according to Fisher et al. (2010), while the C allocation for plant N uptake from soil in clover was then 14 g C g^–1^ N (inverse of CENA_N_, Figure 2B). This result suggests that biological N_2_ fixation by the legume is a relatively C efficient way to acquire N as compared to plant N uptake from soil. Even if the C cost for biological N_2_ fixation is higher than what we assumed (e.g., assuming 10 or 12 g C g^–1^ fixed N, respectively; Fisher et al., 2010), the C cost for biological N_2_ fixation would still be cheaper than or similar to that for N uptake from soil in clover (e.g., C allocation for plant N uptake would then be 13.3 or 12.3 g C g^–1^ N, respectively). Clearly, more work is needed to compare the C cost for biological N_2_ fixation vs. N uptake. Nevertheless, our results may explain why N_2_-fixing plants often compete with non-fixing plants, particularly under the condition of low N availability (Vitousek and Howarth, 1991; Crews, 1999; Menge et al., 2017; Wang et al., 2022).

**FIGURE 3:**
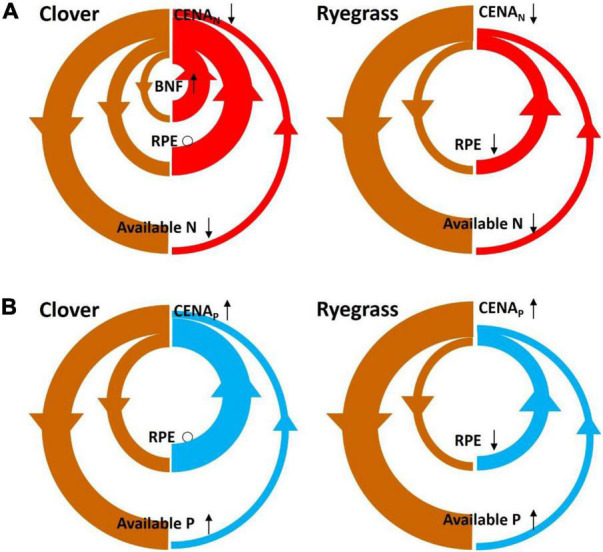
Schematic framework showing how belowground C efficiency for N acquisition (CENA_N_) **(A)** and for P acquisition (CENA_P_) **(B)** vary with soil P availability for clover and ryegrass. Brown arrows, carbon flows; red arrows, nitrogen flows; and blue arrows, phosphorus flows. Thickness of arrows indicates the relative importance of C, N, and P flows, while differences in thickness between carbon and nutrient flows indicate CENA (i.e., relatively thick carbon arrows compared to nutrient arrows indicate a low CENA and vice versa). BNF, biological N_2_ fixation; RPE, rhizosphere priming effect. Black arrows and circles next to CENA_N_, CENA_P_, BNF, RPE, available N, and available P indicate effect of P fertilization (upward arrow, positive; downward arrow, negative; circle, and no effect). For clover, CENA_N_ decreases with P fertilization because the negative effect of P fertilization on N availability outweighs the positive effect on BNF.

Consistent with our hypothesis, clover had a higher CENA_N_ than ryegrass because, as discussed above, less C was required for biological N_2_ fixation than for N uptake from soil. However, CENA_N_ of clover was still higher than ryegrass after we accounted for the C cost associated with biological N_2_ fixation. In contrast to our hypothesis, CENA_P_ was also higher for clover than for ryegrass. Possibly, the greater rhizosphere priming effect on soil organic matter decomposition that we observed for clover by the end of the experiment in another study may contribute to the higher CENA_N_ and CENA_P_ (Lu et al., 2020). By the end of the experiment, available forms of N in soil were extremely low (less than 7 mg N pot^–1^ or 2 mg N kg^–1^ soil), and likely very C expensive to take up by both ryegrass and clover. Therefore, stimulation of soil organic matter decomposition by root exudates and subsequent release of N (and P) for plant uptake may be a very C-efficient way for plants to acquire nutrients from the soil (Figure 3; Wang et al., 2022). Previous studies also suggested that legume species could produce larger rhizosphere priming effects than non-legume species (Cheng et al., 2003; Drake et al., 2013). Alternatively, the higher CENA_P_ in clover than in ryegrass may also be attributed to the tendency of legumes to cause greater acidification in the rhizosphere and exude carboxylates to mobilize and increase concentrations of inorganic P through dissolution or desorption (Hinsinger, 2001; Nuruzzaman et al., 2006).

In contrast to our hypothesis, P fertilization slightly decreased the CENA_N_ in ryegrass and clover, suggesting that P fertilization caused these plants to allocate more belowground C to acquire N from soil. In this study, although biological N_2_ fixation in clover marginally increased with P fertilization (Table 2), the increase of this relatively cheap form of N acquisition was apparently not enough to counter the increase in belowground C allocation for soil N uptake. Possibly, P fertilization may have exacerbated soil N limitation by increasing microbial N immobilization (Blanes et al., 2012; Mehnaz et al., 2019), and thus did not improve belowground C allocation. Indeed, microbial biomass N measured at the end of the experiment was significantly higher with P fertilization (an average of 17%; Lu et al., 2020). In turn, increased microbial N immobilization with P fertilization may thus reduce soil available N, thereby making it more C expensive for plant uptake (Figure 3). It has also been suggested that C allocation could increase to maintain plant N uptake at low N conditions (Brzostek et al., 2014; Perkowski et al., 2021). Our results further imply that plant C allocation belowground for N uptake depends on P availability.

Phosphorous fertilization marginally increased the CENA_P_ for both species, indicating that the P uptake may become somewhat less C expensive with increased soil P availability (Figure 3). This result is consistent with the resource optimization hypothesis that plants allocate more C to acquire limited resources (McMurtrie and Dewar, 2013). Previous studies also found that belowground C investment in P acquisition was more efficient with P fertilization (Ven et al., 2019). We expected that CENA_P_ would increase more for clover than for ryegrass given that growth of the N-fixing clover would be more limited by P than ryegrass (Png et al., 2017). However, we found no support for this (no significant species × P interaction), but we should note that the increases in CENA_P_ with P fertilization were relatively small and only marginally significant. Clearly, more work is needed for an in-depth understanding of the mechanisms underlying the CENA_N_ and CENA_P_ of grasses and legumes under different N and P availabilities.

While we did not investigate interspecific interactions on CENA, the CENA concept may have significant implications for plant community dynamics and competition for nutrients in legume-grass mixtures (Raven et al., 2018; Wang et al., 2022). When legumes have a higher CENA compared to grasses, they may temporarily outcompete grasses for soil nutrients until conditions arise that reduce CENA for legumes more than for grasses (e.g., under drought or high levels of soil N). Changes in CENA with time would also depend on how flexible plants are in switching C allocation toward different nutrient acquisition strategies (Fisher et al., 2010). Furthermore, interspecific competition for nutrients by itself, as well as the transfer of biologically fixed N from legume to grass, could potentially influence the CENA of individual species in grassland mixtures, which could help explain the often observed temporal dynamics in legume and grass abundance in mixed pastures (Ledgard and Steele, 1992). Further research is therefore needed to investigate the role of CENA in plant community dynamics in legume-grass mixtures.

## Conclusion

In our study, we quantified the belowground C allocation and its efficiency for N and P acquisition (CENA_N_ and CENA_P_, respectively). We showed that clover had higher CENA_N_ and CENA_P_ than ryegrass, even after accounting for the relatively low C costs associated with biological N_2_ fixation, possibly because of the distinct rhizosphere priming on soil organic matter decomposition. Furthermore, P fertilization decreased CENA_N_, possibly *via* exacerbating soil N limitation, while P fertilization increased CENA_P_ because plant P acquisition was more efficient with increased P availability. Current modeling studies have indicated that net primary productivity and soil C storage are strongly associated with variation in the belowground C allocation for nutrient uptake (Fisher et al., 2010; Brzostek et al., 2014; Shi et al., 2016). Yet, estimates of CENA_N_ and CENA_P_ are lacking. To the best of our knowledge, this is one of the first studies to provide estimates for these parameters. We acknowledge that our study is based on two plant species only and in one soil type only, and that variation in CENA should be examined across a larger set of plant species, communities, and soil types during a relatively long period. Nevertheless, we believe that better understanding of CENA_N_ and CENA_P_ will help not only improve global C cycling model predictions but also identify management practices to increase yield and fertilizer use efficiency in agricultural systems.

## Data Availability Statement

The original contributions presented in this study are included in the article/supplementary material, further inquiries can be directed to the corresponding author.

## Author Contributions

JL and FD designed the experiment. JL, JY, and CK performed the experiment. JL analyzed the data. JL, LY, PW, WC, and FD wrote the manuscript. All authors contributed to the article and approved the submitted version.

## Conflict of Interest

The authors declare that the research was conducted in the absence of any commercial or financial relationships that could be construed as a potential conflict of interest.

## Publisher’s Note

All claims expressed in this article are solely those of the authors and do not necessarily represent those of their affiliated organizations, or those of the publisher, the editors and the reviewers. Any product that may be evaluated in this article, or claim that may be made by its manufacturer, is not guaranteed or endorsed by the publisher.
